# TEACHING STRATEGIES FOR INTERDISCIPLINARY COLLABORATION THROUGH A PUBLIC HEALTH LENS

**DOI:** 10.1093/geroni/igae098.3115

**Published:** 2024-12-31

**Authors:** Elaine Jurkowski

**Affiliations:** Southern Illinois University Carbondale, Carbondale, Illinois, United States

## Abstract

The Social Determinants of Health (SDOH) have become increasingly important in workforce training for health/medical professionals working with older adults to improve health outcomes. Inter-professional collaboration across disciplines such as primary care medicine, physician assistants, social workers etc. does not occur naturally since educational programs are siloed. However, these disciplines are required to work collaboratively with each other, targeting SDOH’s and patient outcomes. The objective was to promote communication across disciplines and help each discipline understand the roles played in practice with older adults. Three specific strategies to address Interprofessional Education were devised and implemented within a rural context to address the goal of improving collaboration and communication. An educational seminar was conducted using cases and guide questions. A second strategy was through “Student hot-spotting” where students worked with a high-need complex case over the course of a school year weekly, as a team-based approach. A third strategy made use of a weekly medical clinic for team-based patient care with students from medicine, social work, PA, nursing and nutrition. Findings suggest professionals were surprised at what they learned from the other disciplines they were collaborating with. They also learned about community-based resources and improved health outcomes when the lens of public health was in the forefront and an intentional part of the curricula. All participants felt that the chance to collaborate outside of their disciplines strengthened their skills and impact. In conclusion, curricula intentionally devised using a public health lens, in collaboration with other disciplines improves overall collaboration among professionals.

